# Residents' perception and worldview about radon control policy in Canada: A pro-equity social justice lens

**DOI:** 10.3389/fpubh.2022.946652

**Published:** 2022-08-23

**Authors:** Selim M. Khan, James Gomes, Anne-Marie Nicol

**Affiliations:** ^1^Cumming School of Medicine, University of Calgary, Calgary, AB, Canada; ^2^Faculty of Health Sciences, University of Ottawa, Ottawa, ON, Canada; ^3^Faculty of Health Sciences, Simon Fraser University, Burnaby, BC, Canada

**Keywords:** indoor air quality, radon, lung cancer, public health policy, mixed methods, equity, social justice, worldviews

## Abstract

Radon is a potent indoor air pollutant, especially in radon prone areas and in countries with long winters. As the second top lung carcinogen, radon is disproportionately affecting certain population subgroups. While many provinces have taken sporadic actions, the equity issue has remained unaddressed across all policy measures. Attempts to enforce radon guidelines and enact building regulations without considering residents' views have proved ineffective. Research linking residents' radon risk perception and worldviews regarding radon control policy is lacking in Canada. We applied mixed (quantitative and qualitative) methods in a pro-equity social justice lens to examine the variations in residents' risk perception, access to risk communication messages, and worldviews about risk management across the sociodemographic strata. Triangulation of the quantitative and qualitative findings strengthened the evidence base to identify challenges and potential solutions in addressing the health risk through upstream policy actions. Enacting radon control policy requires actions from all levels of governments and relevant stakeholders to ensure equal opportunities for all residents to take the preventive and adaptive measures. Small sample size limited the scope of findings for generalization. Future studies can examine the differential impacts of radon health risk as are determined by various sociodemographic variables in a representative national cohort.

## Introduction

Soil gas radon is a category one human carcinogen (1) and second topmost lung carcinogen after tobacco (2). Non-smoking women, children, Indigenous people, and people of lower socioeconomic groups are disproportionately affected, especially in radon prone areas (3–5). The voluntary guideline of Canadian federal radon policy encourages cooperative control strategies by different level of governments and posits that residents would test and mitigate their houses responsibly (6). Nevertheless, policies in other jurisdictions [e.g., (7)] focus more on creating a system of enforcement requiring member states to implement a wider action plan. Whereas, Canadian federal labor codes are limited to testing and mitigating public buildings, European Union (EU) requires mitigation for workplaces beyond the public buildings and provides incentives for testing and mitigation with intensive local government involvement (8). Although many provinces have acted on this federal radon policy, the most populous provinces—Ontario and Quebec, have lagged behind (9) and the equity issue has remained unaddressed across all policy actions when it comes to testing and mitigating private homes.

A study in Ontario revealed that nearly 73% of lung cancer deaths are attributable to exposure from residential radon at a level below 100 Bq/m^3^ (10). These findings support previous research that there is no threshold or guideline level to harmful exposure to indoor radon and all residents are vulnerable to the exposure of this harmful gas (11, 12), but still the federal guideline level remains 200 Bq/m^3^ and this creates an underestimation of risk by Canadians. Psychological research describes subjective and objective understandings of risk, and a dual cognitive and affective risk perception process (13–16). Social science research determines that the success of any population-level awareness program is dependent on the views and actions of key decision makers at the household level (17). Thus, voluntary implementation of radon guidelines cannot have the desired effect without considering residents' perception and worldviews about the risk.

Although science usually helps decision makers by influencing their perception and beliefs with evidence, the case of radon is unique in the ways people think of risk. The dilemma with radon health risk is that on one hand, decision makers are prone to see the immediacy of a hazard and emergence of a threat—how many people are affecting or are dying in a short period? On the other hand, holders of scientific knowledge often do not understand this type of information need by the decision makers. Explaining this phenomenon often requires interdisciplinary insight that may connect residents' risk perception and worldviews to the decision making by policy makers.

Worldviews are the general social, cultural, and political attitudes, belief and values held by citizens that impact their judgments about complex issues (18). As per Dake [(19), p. 694] worldviews are some “orienting dispositions,” that guide people's responses to the risk. Slovic (18) identified and measured a group of worldviews representing people's attitude toward risk. These are (a) **Fatalism**: People with fatalists' worldview tend to think that whatever happens in life is inevitable. (b) **Hierarchy**: Hierarchists like a top-down administered society where commands flow down from the authorities and obedience flows up the hierarchy. (c) **Individualism**: Such people prefer to do their own business without any interruption from the authority. (d) **Egalitarianism**: People desire a world where powers and means are distributed equally. (e) **Technological Enthusiasm**: People endeavor to stay abreast of the latest development in science and technology and want to get the best out of latest innovations.

We assume that exploring different group of residents' attitudinal risk perception and worldviews would generate evidence for pro-equity policy actions. The scholarship that links science to decision making embraces at least three disciplines such as knowledge translation, risk communication, and decision analysis (20). We adopted an interdisciplinary equity lens and a conceptional policy framework to explicitly talking to critically assessing resident's risk perception and worldviews. **The objective** of this study was to explore residents' perception and worldviews regarding radon health risk and generate evidence that may guide the pro-equity radon control policy in Canada.

## Theories and conceptual policy framework

**Figure 1:**
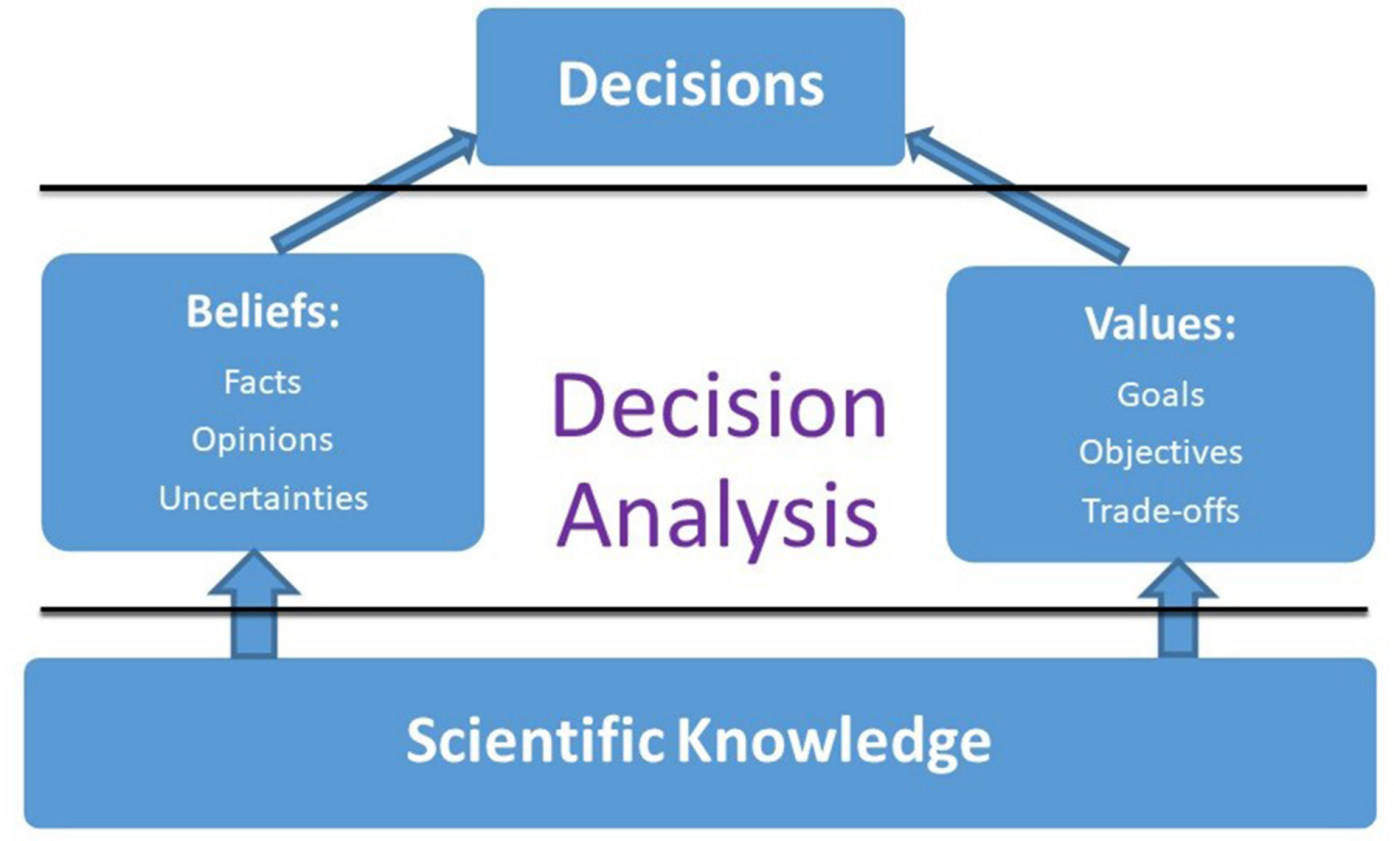
Framework to bridge science and decision making; Winterfeldt (20) (reproduced with permission).

Mainstream decision-making theories assume that decisions are based on two fundamental pillars - beliefs and values (Figure 1). Beliefs involve a decision maker's perception about reality that includes facts, opinions as well as uncertainties. Values indicate a decision maker's sense about something that is worth striving for and accomplishing. These include one's goals, objectives, and associated trade-offs. Although viewed differently by different schools of thoughts, these two concepts theoretically comprise: (a) prescriptive approaches such as expected value theory (21), expected utility theory (22), expected regret theory (23); and (b) descriptive approaches such as prospect theory and cumulative prospect theory (24).

Descriptive prospect theory from cognitive psychology involves the mechanism through which people choose policy options from the probabilistic alternatives that include risk, where the probabilities of outcomes are uncertain (25). In our case of radon, as extensive evidence has removed uncertainties about the outcomes, we examined the first category - prescriptive expected utility theory (22) that has been reformulated using expected regret theory (23). Under certain circumstances, these two formulations yield different results. Where the expected utility theory considers the highest expected value for the best course of action, the expected regret theory looks for a decision that will lead to the least amount of regret. The Decision Curve Analysis (DCA) model in the medical domain (26) found that the expected regret theory is preferable to the expected utility theory as regret is widely recognized as one of the critical decision-making mechanisms, enabling a decision maker to experience consequences of decisions both at the emotional and cognitive level. Nonetheless, these rationalities of expected utility theory go with the people-centered critical social justice theory where testing a home for radon and mitigation pose difficulties for some due to their disadvantaged social positions. They remain unable to ensure the highest expected health value for them, and consequently, suffer greater regret. Application of such critical approaches to social justice refers to the acknowledgment that society is divided and unequal, for differences in race, economic class, gender and education significantly affect their ability to decide in favor of their health (27).

## Methodology

The mixed methodology (28) applied in this study adopts ideas from the interdisciplinary theories in framing the interview protocols and survey instruments that sought to understand multi-level influences on the perception of radon health risk in a Canadian context. The pertinence of such methods was that radon health risk is influenced by a complex set of determinants interacting with each other in various directions (29) that are difficult to explore either by quantitative or qualitative approach alone. We assumed that the pooled analysis would provide a “whole greater than the sum of the parts” [(30), (p. 40)] and the insight gathered would be useful to guide radon control policy.

## Methods

Our Mixed Methods Research (MMR) comprised a complex survey (*n* = 557) with a structured questionnaire including some open-ended questions, and qualitative semi-structured interviews (*n* = 35) including closed and open-ended queries conducted with samples from Ottawa-Gatineau CMA. We tried to decrease bias by involving participants from diverse sociodemographic factions such as homeowner and tenant, male and female, younger adult and elderly, different ethnic groups, levels of education, income and occupational categories.

### Interview protocol and pilot study

Our interview protocol was developed and tested in a mini-pilot study before applying it to the purposefully selected main sample. The questions covered residents' perception and worldviews regarding radon control policy. These qualitative findings guided the development of a survey instrument [for detail: (31)].

### Survey participants

In a cross-sectional design, we surveyed property owners (71%) and tenants (29%). The stratified two-stage cluster random sample selected 140 participants from each of the two cities from public access property rolls and rental agency lists, respectively. The final sample had 557 participants.

### Quantitative measures and variables

The survey consisted of a mixed- closed and open-ended questions. Independent variables included levels of awareness and perception of the risk, worldviews about radon control policy. The outcome variables included intention to test for radon (ordinal), and residents' actual testing and mitigation (binary). The control variables included the socioeconomic determinants such as age, gender, education, occupation, income, race/ethnicity and homeownership or tenancy.

## Analysis

Descriptive, multiple logistic, binary and ordinary regression analyses were conducted with the perception variables and sociodemographic variable to determine differential impacts of radon health risk on population sub-groups, and these included a mixed methods analysis of the residents' worldviews on radon health risk management to generate evidence for pro-equity policy actions (31–33).

## Results

### Demographics of study participants

In the quantitative sample (557), homeowner to tenant participation ratio (71: 29) corresponded to that in the population. The gender ratio could not be ascertained as many residents preferred not to identify their gender. The mean age of our sample was skewed to older group (65+) as they might have spare time to participate in the study and included more homeowners than tenants as tenants have no authority over testing and mitigating the house they live in. Visible minorities were over-represented and Indigenous people under-represented. Most survey takers had some university education but were overrepresented by lower middle-income groups (Table 1).

**Table 1:** Demographics of survey (quantitative) participants.

**Sociodemographic variables**	**Overall**	**Homeowners *n* (%)**	**Tenants *n* (%)**
	**Participation *n* (%)**		
Characteristics	557 (100%)	394 (70.7%)	163 (29.3%)
**Gender**
Male	291 (52.2%)	193 (49%)	98 (60.1%)
Female	224 (40.2%)	170 (43.1%)	54 (33.1%)
Not willing to identify	42 (7.5%)	31 (7.9%)	11 (6.7%)
**Age groups**
18–24 year	83 (14.9%)	51 (12.9%)	32 (19.6%)
25–34 year	58 (10.4%)	42 (10.7%)	16 (9.8%)
35–44 year	59 (10.6%)	42 (10.7%)	17 (10.4%)
45–54 year	85 (15.3%)	63 (16%)	22 (13.5%)
55–64 year	106 (19%)	69 (17.5%)	37 (22.7%)
65 and above	166 (29.8%)	127 (32.2%)	39 (23.9%)
**Race/ethnicity**
European Canadian	375 (67.3%)	271 (68.8%)	104 (63.8%)
Aboriginal Canadian	14 (2.5%)	12 (3.0%)	2 (1.2%)
Visible minorities	120 (21.5%)	76 (19.3%)	44 (27.0%)
Prefer not to answer	48 (8.6%)	35 (8.9%)	13 (8.0%)
**Education**
Elementary	1 (0.2%)	0 (0%)	1 (0.6%)
Some high school	6 (1.1%)	5 (1.3%)	1 (0.6%)
Completed high school	60 (10.8%)	32 (8.1%)	28 (17.2%)
Some Community/technical college/CEGEP			2
	60 (10.8%)	36 (9.1%)	4 (14.7%)
Completed Community/technical college/CEGEP	75 (13.5%)	55 (14%)	20 (12.3%)
Some university	48 (8.6%)	39 (9.9%)	9 (5.5%)
Undergrads	187 (33.6%)	137 (34.8%)	50 (30.7%)
Master, PhD	100 (18%)	74 (18.8%)	26 (16%)
Post doctorate	11 (2%)	8 (2%)	3 (1.8%)
No schooling	3 (0.5%)	3 (0.8%)	0 (0%)
Prefer not to answer	6 (1.1%)	5 (1.3%)	1 (0.6%)
**Income groups**
< $40 K	60 (10.8%)	29 (7.4%)	31 (19%)
$41–75 K	147 (26.4%)	101 (25.6%)	46 (28.2%)
$76–100 K	94 (16.9%)	66 (16.8%)	28 (17.2%)
$101–150 K	106 (19%)	86 (21.8%)	20 (12.3%)
>$150 K	68 (12.2%)	54 (13.8%)	14 (8.6%)
Prefer not to answer	82 (14.7%)	58 (14.7%)	24 (14.7%)

The qualitative study sample consisted of 35 interviewees (Table 2) purposefully selected from the above survey cohort who had some degree of radon knowledge and tested or mitigated their houses for radon. For this reason, the sample was underrepresented by tenants and people from low-income groups.

**Table 2:** Characteristics of qualitative study participants.

**Characteristics**	**Numbers**	**Percentage**
**Gender**
Female	8	23%
Male	27	77%
**Age groups**
18–44	7	20%
45–64	15	43%
65+	13	37%
**Level of education**
High school	3	9%
College	9	26%
Bachelor	11	31%
Graduate	12	34%
**Total household income**
< $40,000	3	9%
Between $41,000 and 75,000	7	20%
Between $76,000 and 100,000	9	26%
Between $101,000 and 150,000	11	31%
Between $151,000 and above	2	5%
Prefer not to answer	3	9%
**Homeownership**
Homeowner	29	83%
Tenant	6	17%

### Variation of risk awareness

From the survey, we found that many residents did not know much about radon whereas the qualitative study provided evidence that a few of them had deeper knowledge about the health issue, indicating their cognitive risk awareness. We were able to explore the reasons behind the variation in risk perception across the population sub-groups. These included (a) **personal** health consciousness, having relevant education or being a lung cancer survivor; (b) **familial**—being concerned about the health of children living in the basement that is the affective or emotional risk awareness; (c) social—interacting with friends and witnessing others in the community diagnosed with or suffering from lung cancer; (d) occupational—taking part in training at one's workplace or learning as part of one's job.

### Variation in risk perception and preventive actions

We sought to understand participants' worldviews of what should be done to raise risk awareness and preventive actions. Upon disaggregation of the quantitative data between homeowners and tenants to explore the variation of perception across the socioeconomic strata (Table 3), gender was significantly correlated with the intention to test for radon both for homeowners and tenants but not with actual testing and mitigation. This accords with past psychological research that women perceive a risk more seriously, but men are pre-emptive in action (15).

**Table 3:** Sociodemographic determinants vs. protection behaviors.

**Sociodemographic variables**	**Intention to test**		**Actual testing**		**Mitigation**	
	**Wald (Sig)**		**Wald (Sig)**		**Wald (Sig)**	
**Characteristics**	**Homeowners**	**Tenants**	**Homeowners**	**Tenants**	**Homeowner**	**Tenants**
Gender	23.18 (0.00)	5.11 (0.02)	1.45 (0.22)	0.44 (0.95)	1.4 (0.23)	NS^*^
Age groups	38.36 (0.00)	7.47 (0.00)	11.41 (0.00)	0.64 (0.42)	8.7 (0.00)	NS
Race/ethnicity	1.3 (0.25)	0.18 (0.66)	1.59 (0.20)	0.18 (0.66)	0.00 (0.95)	NS
Education	5.8 (0.01)	0.65 (0.41)	0.18 (0.67)	0.00 (0.96)	0.17 (0.67)	NS
Income groups	0.37 (0.54)	0.025 (0.87)	0.20 (0.88)	0.01 (0.91)	0.06 (0.80)	NS
Length of year living in current home	0.053 (0.81)	1.2 (0.26)	3.3 (0.06)	2.3 (0.12)	0.03 (0.85)	NS
Living space in the basement	4.5 (0.03)	0.68 (0.40)	0.34 (0.55)	0.17 (0.67)	1.8 (0.17)	NS
Consider radon a threat to your or family's health	5.7 (0.01)	0.76 (0.01)	0.05 (0.94)	1.78 (0.18)	0.07 (0.78)	NS
Anyone from HH diagnosed with lung cancer	5.3 (0.02)	0.37 (0.54)	0.34 (0.56)	0.08 (0.76)	0.02 (0.88)	NS
Worldviews	1.2 (0.26)	3.1 (0.07)	2.13 (0.14)	0.94 (0.33)	0.62 (0.43)	NS

Multiple Logistic regression: Method = Forward Stepwise.

* NS, no statistics, Wald, Wald Chi-Squared Test; Sig, Significant level set to *p* ≤ 0.05.

Age was associated with the intention to test for radon both for homeowners and tenants; when it came to testing and mitigating homes, it was significant for the homeowners but not for the tenants. Qualitative analyses revealed that homeowners were comparatively older, had children and they ranked higher in terms of care for health. We found no correlations of ethnicity with either intention to test or protection behaviors for homeowners or tenants.

Education was significantly correlated with the intention to test for radon in the case of homeowners but not tenants. An exploration of the qualitative data found that older homeowners invested more time to understand the risk. Alternately, most tenants knew about the risk either from their workplace or from informed colleagues. Income was not correlated with the intention to test or protective behaviors in either homeowners or tenants. These findings contradicted the qualitative result as residents who tested and mitigated homes for radon were generally from higher income brackets.

### Variations in worldviews about policy actions

We asked participants to provide their world views on how to help raise awareness and take action on radon. The results showed five distinct types of worldviews:

#### Fatalism

Some residents think the exposure to radon is natural and so the consequence is preordained. They typically feel to have very little control over the risk to their health. A very few residents possess such a worldview. As per one, “Its naturally occurring and it happens everywhere. We can do very little about it.” (SP13). Another said “No, I don't think (it's a risk) right now because I live in a new home” (SP2).

#### Hierarchy

Majority of residents are on the side of a top-down administered societal order and they wanted regulations and instruction to come down from the authorities. They think, people will comply when recommendations about the health risk come from experts in the field. They stressed on the role of public health agencies. Many emphasized on joint efforts in terms of radon health risk communication to be made by all levels of governments such as …“municipal, provincial and federal” (SP15). They sought engagement of “all stakeholders who may be involved in this issue” (SP2). These should include private sectors (e.g., home builders) and associations (e.g., Cancer Society) along with the government. Their worldviews entailed the using of multitude of public media as different age and education groups of people have varied interests and access to media. They added that authentic government health agencies such as Health Canada has to launch effective health education programs employing different print (e.g., newspapers), audio (radio ads) and visual (tele-script) media. Other residents' view was to employ a mix of media strategies such a figurative ad in television that illustrates the effects of exposure to radon that would be verily understood by residents (SP25). While other views were to send out risk communication messages in the residential mailboxes through illustrative pamphlets as different utility companies do, publishing promotion articles in local newspapers- print or online or airing realistic programs on television or any combination of these approaches (SP29).

#### Individualism

A few residents had the worldview that this health risk relates to individual's duty of care, and they associated person's unfavorable behaviors at the root of this risk. Therefore, they stressed on individual responsibilities without much interruption from the government or any other authorities. As per them, it is an individual issue as it depends on many personal behavioral and life-style aspects such as not opening windows often, smoking indoors etc., thus, the responsibility to solve the problem goes to the individuals, not to the society (SP35). While others' think although it is an individual issue and the onus goes primarily to the individuals, the government cannot evade the responsibility of providing support with the right information and assistance where warranted (SP16).

#### Egalitarianism

Many of our residents demonstrated egalitarian worldview that is in line with the vision of social justice. Some call it a “share responsibility” (SP20, SP34). They view this as this is an overall societal issue, not personal as it spans from the coast to coast (SP7). Others viewed the risk as a societal issue because it affects every person without discrimination (SP30). The policy recommendation from this group of residents was that primarily the governments are responsible for the dissemination of health risk information where the homeowners should take first step to test (SP11, SP12). Many others held a mixed worldviews as they think whenever a homeowner cannot bear the cost of mitigation, government support should be made available (SP11, SP12).

#### Technological enthusiasm

Some of our residents showed advanced technical knowledge, they read scientific articles on the health risk, got training from their workplace and have taken proactive action in testing their houses for radon and also remediated and bought continued radon monitoring devices. This early adopters' group, as usual consists of very few residents but they had diverse views as manifested by the suggestions they offered.

Among other views from residents included (i) changing in the culture of practice at the clinical and family physician's service settings (SP1); (ii) mobilizing the role of citizenry to raise public awareness through social and political movement (SP20), especially by the people in radon prone areas (SP13); (iii) providing some kind of incentives such as free test kits, tax rebate, subsidies etc., that are to be enacted through legislation or by law (SP27); (iv) making radon testing an essential clause in the property sales agreement (SP32); (v) mandating radon test through obligatory building codes where the onus goes to the builders rather than individual homeowners (SP24); according to some, if Carbon Monoxide detector can be made mandatory; then, why not a radon detector (SP30) that is killing more people than the former.

## Discussion

Although not representing the perspectives of all Canadians, the identified worldviews illustrated diverse notions on how the risk is perceived and how they actions are to be taken by different level of stakeholders. We noted the limited knowledge about radon in a few participants led them to have fatalistic worldviews and their worldviews about risk were not always based on sound science and decisions based on their ideas could sometime be potentially harmful. This indicates that current radon health communication programs are not meeting the objective of health communication for most of the residents. Whereas, individualistic, egalitarian as well as mixed worldviews guided to identify the effective communication strategies and policy directions. The varied impacts of different mass media and variations in accessing by different age-groups of people- such as elder residents' access to health message through newspapers and radio whereas younger ones' comparatively more access through internet and social media. These indicate the need for applying a range of health risk communication strategies. This finding corresponds with previous study conducted by Nicol et al. (38). Our findings supported the constructs of DCA model as residents' worries about children living in the basement lead them to the critical decision-making as they were moved by both the emotional and cognitive perception of the risk (26).

Residents with individualistic worldview, think that the risk is an individual health issue and consider the responsibility for testing and mitigating to lie with the individuals, while the hierarchists believe that different level of governments and responsible agencies are responsible to address the issue applying various strategies. The egalitarian worldview holders consider radon health risk to be a societal issue as many residents are not in a position to decide or financially able to control the risk either because of information deficiencies or due to economic reasons. Yet other residents view the risk as a shared obligation for both the homeowners-tenants as well as authorities. There are people with enthusiasm in technology, advanced knowledge and financial ability who have already taken the necessary prevention actions. Some are enthusiastic in raising awareness through social movement that can build an overall public opinion and such power of politics can push the appropriate authorities to play their roles. The suggestions gathered from residents' worldviews include providing incentives, mandating action through targeted mass media campaigns and enacting building codes, enacting public health regulations and building codes through different policy levels actions by the multi-tier government systems in Canada.

Through a review of radon laws and policies in Canada and abroad, Quastel et al. (36), identified the gaps and variations in the adoption, enforcement and modernization of the building codes in different provinces. Whereas, building codes apply throughout the province, the radon-related provisions allocated only by geographical consideration in Ontario (9). Although processes are underway to updating building codes, mandating building design and construction, and testing houses for radon as a basic requirement (39), a pro-equity approach to cover the most affected population subgroups is not visible. Similarly, Quebec Construction Code (QCC) was amended in 2016. However, it kept the previously set reference level of 800 Bq/m^3^ of 1987 (40). Synthesizing the best practices of radon policies in Canada and six European countries—Denmark, Finland, Sweden, the UK, Norway and Switzerland, the report (36) identified the same determinants as we explored, and the strategic policy propositions pinpointed in the report are relevant to our project. As they observe, Canadian federal systems of governance, radon-related regulation, laws and policies have the form of a shared responsibility. Federal guideline cannot require provincial or territorial actions, it only provides a comprehensive policy direction and encourages coordinated efforts. Whereas, the Council of European Union's (7) basic safety standards directive can require member states to address radon in both private and public buildings by developing radon action plans and a system of enforcement. Canadian governments are not using either spending or taxation powers to financially support residents of lower income group to mitigate radon while such assistance is available in countries like Norway, Sweden, Switzerland and the UK. Such a provision could be useful for low-income tenants and First Nations people living in reserve housing. The Federal-Provincial-Territorial Radiation Protection Committee, who holds the mandate to advance harmonization of radiation protection practices and standards across the country, could do more through enacting regulations such as promoting radon training for inspectors who look after compliance with building codes, occupational health and safety and public health guidelines because they have public health regulatory powers. These guidelines can support enacting building and labor codes, real-estate warranty programs, workplace and occupational safety laws, residential tenancies and occupier's liability, public health requirements of school and childcare facilities.

We want to add that the implementation of radon health risk management policy requires a satisfactory ethical reflection. The most important ethical principle of radon health risk management relates to social justice. We have noted the issue of unequal distribution of the risk both in terms of differences in residents' risk perception and their ability to manage it. Keeping these in consideration, radon health risk management strategies should:

(a) Design a fair, transparent and participatory process of decision-making so that it remains unbiased and objectively covers all the vulnerable houses irrespective of their power to pay for the testing and mitigation.

(b) Look for solutions that can be implemented with the limited risk management resources to maximize the benefit for those who are in need.

(c) Any strategy adopted should not increase the health equity gap instead close it as far as possible.

In light of the above, our recommendations are as follows (Table 4):

**Table 4:** Re commendations.

**Area of intervention**	**Recommendation**
**Policy level**	1) A national radon testing and mitigation framework be established under the auspices of Health Canada and followed thoroughly by agencies at all levels of governmental in partnership with the non-governmental initiatives. 2) Radon testing and mitigation can be well-integrated with the energy efficiency housing program. Indeed, there is an even greater need to address radon where initiatives exist to increase airtightness in buildings (34 ).
**Risk communication**	There should be state-of-art public service risk communication materials in the mass media using video scripts and infographics to demonstrate the risk in real-life scenarios, for example, how children and women are exposed to radon gas living in the basement and passing most of the time indoors (3, 35).
**Key stakeholders engagement**	1) Other than NRC, custodian of National Building Codes, partners with similar mandates such as occupational health and safety (OHS), real estate transactions and home warranty programs, occupiers' liability, residential tenancies, childcare and schools and public health agencies could be engaged in implementing radon control policy. 2) Health professionals, particularly physicians and healthcare nurses, can engage with patients who smoke or are exposed to second-hand smoking to raise awareness about the increased risk of lung cancer from the synergistic effect of tobacco smoking. 1) Health Canada can engage public health professionals including public health nurses and dentists, environmental health professionals, public health inspectors through their departmental or organizational mandates to support raising public awareness through their professional activities.
**Testing and mitigation**	1) While the small initial testing cost could go to the homeowners, the claim to grant a percentage of the mitigation cost in the form of tax credit based on the total household income could be considered upon submitting the radon test results with a quote for mitigation from certified radon professional. This claim has been raised from multiple fronts (36, 37).
	2) It should be made mandatory to test and mitigate by certified radon professional to ensure value of the money spent as well as effectiveness of the mitigation system.
**Building codes**	Measures to protect Canadians should include 1) Incorporating radon testing and mitigation requirements by constructors in the National Building Codes. 2) Updating radon guidelines for both workplace and residential buildings in accordance with the latest scientific development 3) Mandating testing and mitigation in all properties including schools, care homes, day care centers, hospitals, and other public and private buildings before granting license, selling and leasing.
**Energy efficiency**	Making building energy efficient must be coupled with testing buildings for radon before and after the procedures and mitigate where necessary.

## Conclusion

This mixed methods study identified differential radon risk perceptions and worldviews about the risk management held by various population sub-groups in Canada. From a population health perspective, we examined the how these perceptions and worldviews differ across the demographic and socioeconomic determinants of health that directly affect residents' decision-making. In addition, our study identified the challenges that surface while addressing the health risk and determined the leverage points of radon health risk communication. Concerted efforts are needed from all levels of governments as well as relevant stakeholders to address these determinants through pro-equity policy actions to ensure preventive and adaptive measures are taken for residents who need supports so that the gaps in health equity are reduced. Our study is limited by small sample sizes; so, the future studies need to address the determinants in a representative national cohort.

## Data availability statement

Data are available from the corresponding author on reasonable request.

## Ethics statement

The studies involving human participants were reviewed and approved by the University of Ottawa's Health Sciences and Science Research Ethics Board (REB) approved the study and data collection protocols (file number: H10-17-03) on December 13, 2017. The patients/participants provided their written informed consent to participate in this study.

## Author contributions

SMK conducted the survey, qualitative interviews, prepared the draft with close collaboration and discussion and maintained close communication with JG. SMK and JG jointly designed the study. A-MN critically reviewed the manuscript from radon policy and regulatory perspectives, provided input, received and added input and based on that prepared the manuscript, got final authorization for publication, reviewed the final document, and contributed to the designing of the paper. All authors contributed to the article and approved the submitted version.

## Funding

This work was supported by McLaughlin Centre for Population Health Risk Assessment, University of Ottawa (#389969) and Telfer School of Management, University of Ottawa (#602733).

## Conflict of interest

The authors declare that the research was conducted in the absence of any commercial or financial relationships that could be construed as a potential conflict of interest.

## Publisher's note

All claims expressed in this article are solely those of the authors and do not necessarily represent those of their affiliated organizations, or those of the publisher, the editors and the reviewers. Any product that may be evaluated in this article, or claim that may be made by its manufacturer, is not guaranteed or endorsed by the publisher.
